# Tumor Treating Fields modulate apoptotic and immune programs in T-cell acute lymphoblastic leukemia cell lines

**DOI:** 10.3892/etm.2026.13195

**Published:** 2026-05-29

**Authors:** Jinju Heo, Myonggeun Yoon

**Affiliations:** 1 Department of Biomedical Engineering, Korea University, Seoul 02841, Republic of Korea; 2 R&D Center, FieldCure Ltd., Seoul 02852, Republic of Korea

**Keywords:** T-cell acute lymphoblastic leukemia, Tumor Treating Fields, alternating electric fields, apoptosis, mitochondrial stress, immunomodulation

## Abstract

T-cell acute lymphoblastic leukemia (T-ALL) is a highly aggressive hematologic malignancy with limited treatment options for relapsed or refractory disease. Tumor Treating Fields (TTFields), low-intensity alternating electric fields that disrupt mitotic spindle assembly, have been clinically determined in numerous solid tumors, yet their effects in hematologic malignancies remain incompletely understood. In the present study, the cellular responses of T-ALL cells to TTFields were examined. Jurkat and MOLT-4 cells were exposed to 150 kHz and 0.7 V/cm fields for up to 96 h. TTFields treatment progressively suppressed cell proliferation, induced G_2_/M-phase arrest, altered cell-cycle distribution, increased apoptotic fractions and increased late-apoptotic fractions. TTFields exposure also increased side-scatter intensity (indicative of increased cellular granularity or internal complexity) and MitoTracker green fluorescence in both cell lines. Intracellular ATP levels were reduced in Jurkat cells, whereas no significant change was observed in MOLT-4 cells. In parallel, transcriptional analyses revealed downregulation of CD69 and IL-2 and upregulation of RELA proto-oncogene, NF-κB subunit and CBL proto-oncogene B. Collectively, these results demonstrate that TTFields induces cytostatic and apoptotic effects accompanied by measurable structural, bioenergetic and transcriptional alterations in T-ALL cells, supporting further investigation of TTFields as a physical therapeutic approach for hematologic malignancies.

## Introduction

T-cell acute lymphoblastic leukemia (T-ALL) is an aggressive hematologic malignancy, accounting for 10-15% of pediatric and ≤25% of adult ALL cases (1). Although first-line treatment outcomes have improved across the past decades, the prognosis of relapsed or refractory T-ALL remains limited, with reduced salvage options and frequent reports of drug resistance and treatment-related toxicity (2). The molecular heterogeneity of T-ALL, driven by diverse mutations such as NOTCH-1, IL-7 receptor and PTEN, further complicates the establishment of standardized therapeutic strategies (3). These challenges underscore the urgent need for novel therapeutic modalities that operate through mechanisms distinct from conventional chemotherapy and targeted therapy.

Among emerging non-pharmacological therapeutic modalities, alternating electric fields, also referred to as Tumor Treating Fields (TTFields), have gained marked attention. TTFields disrupt the alignment of polar intracellular structures such as microtubules during mitosis, leading to mitotic arrest and apoptosis (4). Clinically, TTFields has demonstrated meaningful survival benefits across a number of solid tumors. In glioblastoma, the EF-14 phase III trial showed that adding TTFields to maintenance temozolomide administration improved median overall survival (OS) from 16.0 to 20.9 months (5). In metastatic non-small cell lung cancer, the LUNAR phase III trial similarly extended OS (13.2 vs. 9.9 months) and resulted in Food and Drug Administration approval for both glioblastoma and non-small cell lung cancer (6). A phase III study in locally advanced pancreatic cancer (PANOVA-3) also reported a statistically notable survival benefit (7), supporting the broader clinical applicability of TTFields in solid tumors.

In parallel, experimental evidence has begun to support the efficacy of electric-field exposure in hematologic systems. In U937 acute myeloid leukemia cells, exposure to a 200 kHz, 0.75 V/cm field (alone or combined with daunorubicin), markedly inhibited proliferation and increased DNA damage and apoptosis, while sparing normal lymphocytes (8). Similarly, in a study using the blood samples of patients with coronavirus disease 2019, electric-field stimulation suppressed hyperactivated lymphocyte proliferation and cytokine secretion without affecting quiescent immune cells (9), suggesting that electric-field stimulation can regulate immune cell activation status, cytokine production and systemic immune signaling. Recent simulation-based research has further demonstrated that electric field intensity within bone marrow microenvironment can be optimized by adjusting electrode geometry, applied voltage and field frequency (10). These findings collectively suggest that electric-field exposure may exert selective effects on malignant hematopoietic cells while preserving normal immune populations, providing a theoretical basis for extending TTFields therapy to hematologic contexts.

Despite these emerging insights, to the best of our knowledge, no comprehensive study has yet examined how TTFields affects the biological and immunological behavior of T-cell leukemia. The present study aimed to address this gap by characterizing the overall cellular and immune responses of T-ALL under TTFields exposure. By elucidating the key mechanisms through which TTFields influence leukemic T-cells, the present study sought to provide a conceptual framework for extending electric field-based therapies from solid tumors to hematologic malignancies.

## Materials and methods

### Cell culture

Human T-ALL cell lines, Jurkat (cat. no. 40152) and MOLT-4 (cat. no. 21582), were obtained from the Korean Cell Line Bank. Cells were cultured in RPMI-1640 medium (Gibco; Thermo Fisher Scientific, Inc.) supplemented with 10% FBS (Gibco; Thermo Fisher Scientific, Inc.), 1% penicillin-streptomycin (Gibco; Thermo Fisher Scientific, Inc.) and sodium carbonate (final concentration: 1.5 g/l) to maintain pH stability during incubation. Cells were maintained in T-25 culture flasks (SPL Life Sciences) at 37˚C in a humidified incubator with 5% CO_2_, under static (non-shaking) conditions. Culture medium was replenished every 2 days by adjusting the cell density to 1x10^5^ cells/ml with fresh complete medium. CCD-112CoN (human colon fibroblast; CRL-1541™; American Type Culture Collection) was used as a representative non-malignant human cell line and cultured according to the supplier's instructions.

### TTFields treatment

Electric fields were applied using a custom-built setup consisting of a function generator (cat. no. AFG-2112; Good Will Instrument Co., Ltd.; an amplifier A-303; A.A. Lab Systems Ltd.) and a sterilized insulated wire-based electrode system. Cells were seeded at a density of 1x10^5^ cells/ml in a 10 cm culture dish containing 10 ml complete RPMI medium. Then, two sterilized insulated wires were positioned 7 cm apart at the center of the dish to ensure uniform field distribution. Electric field stimulation was applied at 150 kHz with a field intensity of 0.7 V/cm for 24-96 h. Control groups were cultured under the same conditions without electric field exposure. All cultures were maintained in a humidified 5% CO_2_ incubator at 37˚C without agitation. CCD-112CoN cells were exposed to TTFields under the same experimental conditions as T-ALL cell lines.

### T-cell activation and stimulation

After completion of TTFields exposure, cells were stimulated with the eBioscience™ Cell Stimulation Cocktail (500X; Thermo Fisher Scientific, Inc.) for 2 h before subsequent analyses. This stimulation, performed after electric field treatment, induced T-cell receptor (TCR)-mediated activation and cytokine gene transcription, providing a reference condition for evaluating the effects of TTFields on T-cell activation.

### Cell viability assay

To evaluate the effect of TTFields on cell viability, cells were cultured for up to 96 h with or without electric field stimulation. At 24 h intervals, aliquots of cells were transferred into 96-well plates. Water-Soluble Tetrazolium-8 Cell Counting Kit reagent (cat. no. QM2500; Biomax) was then added to each well, followed by incubation for ≥1 h. Metabolic activity, as an indicator of cell viability, was quantified by measuring absorbance at 450 nm.

### Flow cytometry for T-cell activation markers

After TTFields treatment and stimulation, cells were incubated on ice for 15-20 min in the dark with fluorochrome-conjugated anti-CD69 antibody (cat. no. 310909; BioLegend, Inc.). Stained cells were washed, resuspended in FACS buffer (PBS containing 2% FBS) and analyzed using the BD Accuri™ C6 Plus flow cytometer (BD Biosciences). Data were processed using FlowJo™ software (v10.6.2; FlowJo, LLC). The mean fluorescence intensity (MFI) was defined as the MFI of the gated cell population, which is less sensitive to outliers and therefore suitable for skewed fluorescence distributions.

### Forward scatter (FSC)/side scatter (SSC) analysis

Cells were analyzed using flow cytometry (BD Accuri™ C6 Plus flow cytometer; BD Biosciences) to assess changes in cell morphology. FSC-area (A) and SSC-A signals were acquired. FSC-A reflects the relative cell size based on forward light scattering, whereas SSC-A reflects intracellular granularity or structural complexity based on side-scattered light. Data were analyzed using FlowJo™ software (v10.6.2; FlowJo, LLC).

### MitoTracker (fluorescence) staining

For mitochondrial staining, cells were incubated with MitoTracker™ Green FM (cat. no. M7513; Invitrogen; Thermo Fisher Scientific, Inc.) according to the manufacturer's instructions. Briefly, cells were incubated with MitoTracker™ Green FM at a final concentration of 100 nM for 30 min at 37˚C in the dark. Following incubation, cells were washed with PBS and resuspended in PBS for analysis. Fluorescence signals were acquired using the BD Accuri™ C6 Plus flow cytometer (BD Biosciences) and data were analyzed using FlowJo™ software (v10.6.2; FlowJo, LLC).

### Apoptosis assay

Apoptosis was evaluated using the Annexin V-FITC/PI Apoptosis Detection Kit (cat. no. ab14085; Abcam). Following TTFields treatment, cells were washed with cold PBS and resuspended in binding buffer. Cells were stained with Annexin V-FITC and PI according to the manufacturer's protocol and incubated for 15 min at room temperature in the dark. Stained cells were analyzed immediately using flow cytometry. Early and late apoptotic populations were quantified based on Annexin V and PI double staining.

### Cell cycle analysis

After TTFields treatment, cells were harvested, washed twice with PBS and fixed in cold 70% ethanol at -20˚C overnight. Fixed cells were washed, treated with RNase A (100 µg/ml) and stained with PI (50 µg/ml) in PBS for 30 min at room temperature. Cell-cycle distribution was analyzed using the BD Accuri™ C6 Plus flow cytometer (BD Biosciences) and its accompanying software to determine the proportions of cells in sub-G_1_, G_0_/G_1_, S and G_2_/M phases.

### Intracellular ATP measurement

Intracellular ATP levels were measured using the CellTiter-Glo^®^ Luminescent Cell Viability Assay (Promega Corporation). After TTFields exposure, viable cells were counted using a hemocytometer with trypan blue exclusion under a light microscope and 5,000 live cells/well were seeded into white opaque 96-well plates. Following 24 h stabilization, 100 µl CellTiter-Glo^®^ reagent was added to each well and luminescence was measured using a microplate reader (Titertek-Berthold) with Mikrowin 2000 software (version 4.0; Berthold Technologies GmbH & Co. KG).

### RNA extraction and reverse transcription-quantitative PCR (RT-qPCR)

Total RNA was isolated using the Hybrid-R™ RNA Purification Kit (GeneAll Biotechnology Co., Ltd.) following the manufacturer's protocol. The quantity and purity of the extracted RNA were determined using a NanoDrop™ 2000 spectrophotometer (Thermo Fisher Scientific, Inc.). For cDNA synthesis, 1 µg total RNA was reverse-transcribed using the AccuPower^®^ CycleScript RT PreMix (dT20) kit (Bioneer Corporation) according to the supplier's instructions. RT-qPCR was carried out using the RealAmp™ 2X qPCR Master Mix (cat. no. 801-051; GeneAll Biotechnology Co., Ltd.) on a StepOnePlus™ Real-Time PCR System (Applied Biosystems^®^; Thermo Fisher Scientific, Inc.). The thermocycling conditions were as follows: Initial denaturation at 95˚C for 10 min, followed by 40 cycles of denaturation at 95˚C for 15 sec, annealing at 60˚C for 30 sec and extension at 72˚C for 30 sec. Relative mRNA expression levels were calculated using the comparative Cq (2^-ΔΔCq^) method (11). GAPDH and ribosomal protein L13a (RPL13A) were used as internal reference genes and their geometric mean was employed for normalization. The target genes analyzed in the present study were CD69, IL-2, RELA proto-oncogene (NF-κB subunit), thymocyte selection associated high mobility group box (TOX) and CBL proto-oncogene B (CBLB), with GAPDH and RPL13A used as reference genes. The primer sequences were as follows: CD69 forward, 5'-GCTGGACTTCAGCCCAAAATGC-3' and reverse, 5'-AGTCCAACCCAGTGTTCCTCTC-3'; RELA forward, 5'-TGAACCGAAACTCTGGCAGCTG-3' and reverse, 5'-CATCAGCTTGCGAAAAGGAGCC-3'; IL-2 forward, 5'-AGAACTCAAACCTCTGGAGGAAG-3' and reverse, 5'-GCTGTCTCATCAGCATATTCACAC-3'; TOX forward, 5'-CGCTACCTTTGGCGAAGTCTCT-3' and reverse, 5'-CTGGCTCTGTATGCTGCGAGTT-3'; CBLB forward, 5'-TGCCGATGCTAGACTTGGACGA-3' and reverse, 5'-TGATGTGACTGGTGAGTTCTGCC-3'; GAPDH forward, 5'-GTCTCCTCTGACTTCAACAGCG-3' and reverse, 5'-ACCACCCTGTTGCTGTAGCCAA-3'; RPL13A forward, 5'-CTCAAGGTGTTTGACGGCATCC-3' and reverse, 5'-TACTTCCAGCCAACCTCGTGAG-3'.

### Statistical analysis

All experiments were independently repeated at least three times. Data are presented as the mean ± SD. Statistical analyses were performed using GraphPad Prism (version 8.0; Dotmatics). Normality was assessed using the Shapiro-Wilk test. For comparisons between two groups, an unpaired two-tailed Welch's t-test was applied to account for potential inequality of variances. For comparisons involving ≥3 groups with a single factor, one-way ANOVA followed by Dunnett's or Tukey's post hoc test was used, as appropriate. Two-sided P<0.05 was considered to indicate a statistically significant difference.

## Results

### TTFields suppress T-ALL cell proliferation in a time- and intensity-dependent manner

**Figure 1:**
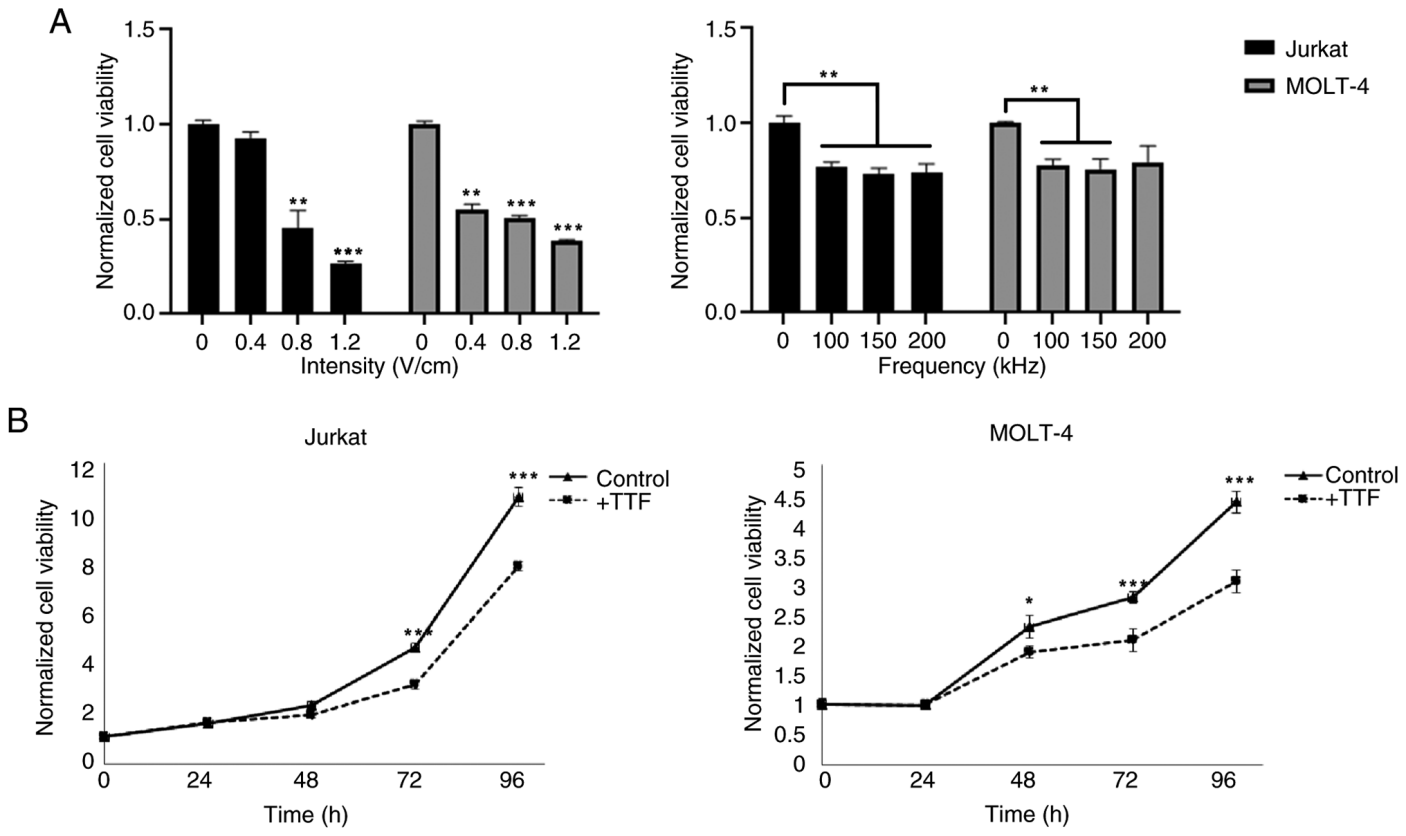
TTFields reduce viability of T-cell acute lymphoblastic leukemia cells in an intensity- and time-dependent manner. (A) Normalized cell viability of Jurkat (black) and MOLT-4 (gray) cells after exposure to 0.0-1.2 V/cm TTFields. Jurkat exhibits minimal change at 0.4 V/cm but sharp reductions at ≥0.8 V/cm; MOLT-4 declines across all intensities. The right panel shows cell viability across the tested frequency range, with no marked frequency-dependent differences observed. (B) Time-course of live-cell counts (0-96 h) under control vs. TTFields. Error bars represent the mean ± SD of three independent experiments. ^*^P<0.05, ^**^P<0.01 and ^***^P<0.001. TTFields/TTF, Tumor Treating Fields.

Jurkat and MOLT-4 cells exposed to increasing electric-field intensities exhibited a significant dose-dependent reduction in cell viability (Fig. 1A, left). Jurkat cells exhibited relative resistance at lower field intensities, whereas MOLT-4 cells displayed a more progressive decline across the tested intensity range. When TTFields was applied at varying frequencies, both cell lines demonstrated reduced viability compared with controls, with no significant frequency-specific differences within the tested range (Fig. 1A, right). Based on these findings, 150 kHz was selected as a representative frequency for subsequent experiments. Using this selected frequency and an electric-field intensity of 0.7 V/cm, time-course analyses further demonstrated that TTFields exposure slowed cell accumulation in both Jurkat and MOLT-4 cells, with proliferation suppression becoming more pronounced at later time points (Fig. 1B). Accordingly, a TTFields condition of 150 kHz at an electric-field intensity of 0.7 V/cm was selected for subsequent experiments, as it was technically feasible and consistently produced reproducible cytostatic effects in both T-ALL cell lines. To evaluate whether these effects were specific to malignant cells, a non-malignant human fibroblast cell line (CCD-112CoN) was exposed to TTFields under the same experimental conditions. TTFields exposure did not significantly affect metabolic activity or cell number in CCD-112CoN cells (Fig. S1).

### TTFields exposure alters cell-cycle distribution and induces apoptosis in T-ALL cells

**Figure 2:**
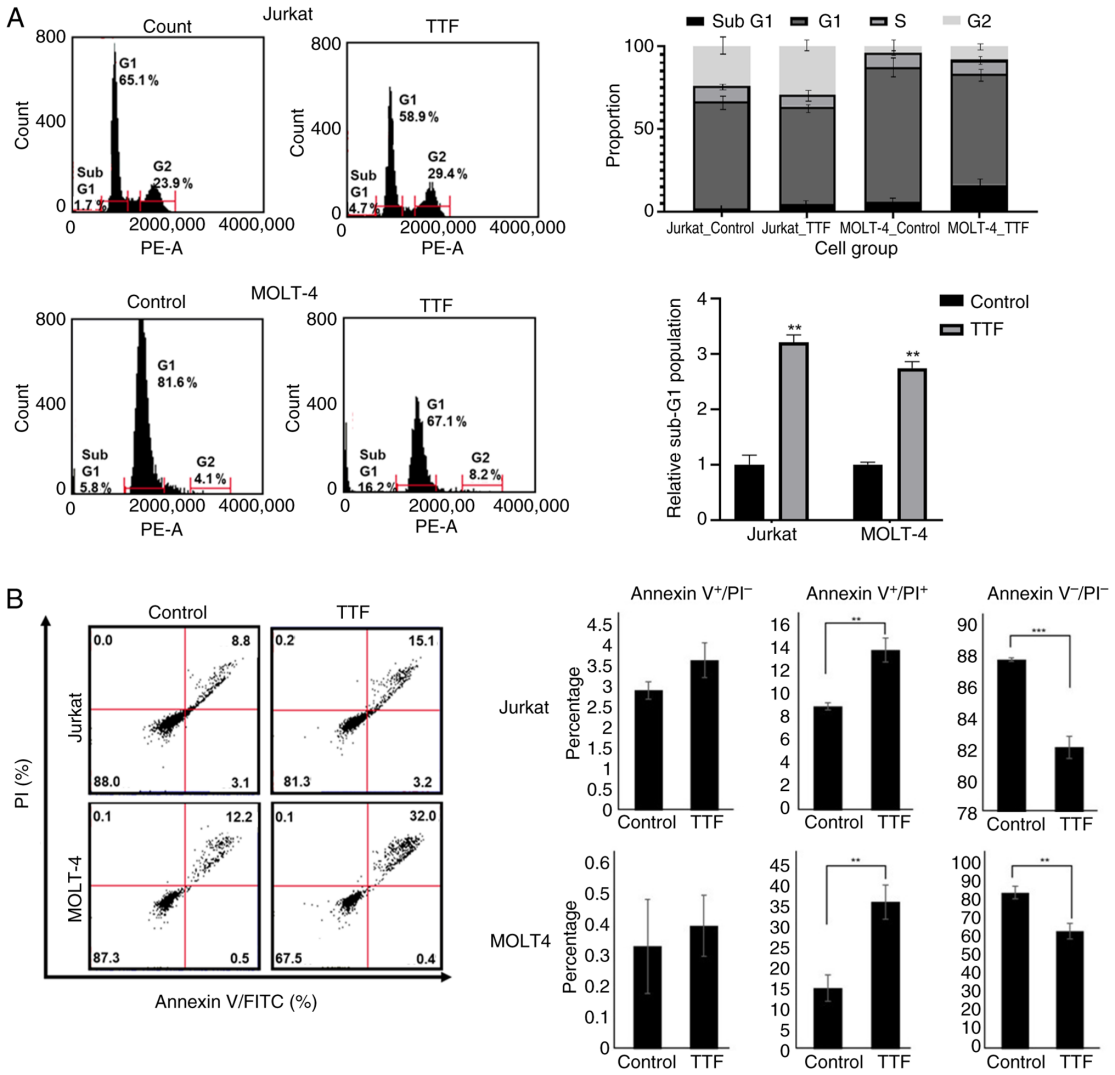
Effect of TTFields on cell-cycle distribution and apoptosis in T-ALL cell lines (A) Cell-cycle analysis showing an increased sub-G_1_ fraction and a decreased G_1_ phase population following TTFields treatment, indicating increased apoptotic fractions. (B) Annexin V/PI staining demonstrates elevated late-apoptotic cell populations and reduced viable fractions. Data are presented as the mean ± SD from three independent experiments. ^**^P<0.01 and ^***^P<0.001. TTFields/TTF, Tumor Treating Fields; PE-A, phycoerythrin-area; Con, control.

Given that TTFields are known to disrupt microtubule polymerization dynamics and impair cytokinetic progression, the present study examined whether T-ALL cells exhibit corresponding changes in cell cycle progression and apoptosis. Flow cytometric profiling showed that TTFields altered cell-cycle distribution, characterized by a reduction in the G_1_ population and modest changes in G_2_/M distribution (Fig. 2A). Apoptosis analysis revealed elevated late-apoptotic fractions following TTFields exposure, accompanied by a decrease in viable cells, whereas the proportion of early apoptotic cells (Annexin V-positive/propidium iodide-negative; lower right quadrant in Fig. 2B) did not show a statistically significant change compared with the control group. These findings indicate that TTFields promote both mitotic arrest and apoptosis in T-ALL cells.

### TTFields increase intracellular granularity and alter mitochondrial-associated readouts in T-ALL cells

**Figure 3:**
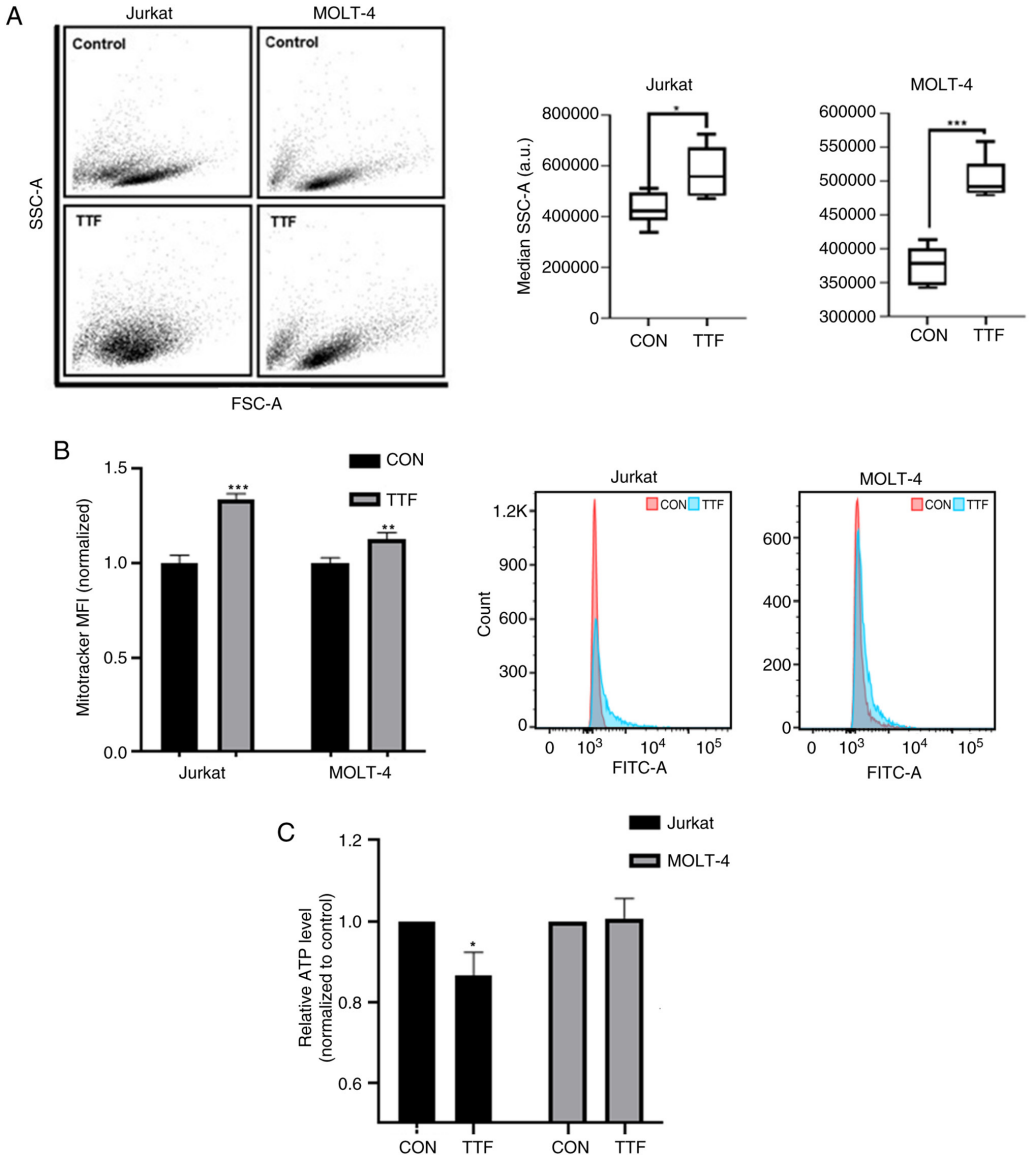
TTFields increase cellular granularity and mitochondrial fluorescence in T-cell acute lymphoblastic leukemia cell lines. (A) Representative FSC/SSC plots and quantification of side-scatter mean fluorescence intensity (median SSC-A values). FSC-A associates with cell size, while SSC-A reflects intracellular complexity such as organelle content and granularity. (B) MitoTracker green fluorescence intensity was normalized to control values and was significantly elevated in both Jurkat and MOLT-4 cells following TTFields treatment. (C) Intracellular ATP levels measured using the CellTiter-Glo luminescence assay and normalized to control values. Data are presented as the mean ± SD from three independent experiments. ^*^P<0.05, ^**^P<0.01 and ^***^P<0.001. TTFields/TTF, Tumor Treating Fields; SSC, side scatter; FSC, forward scatter; A, area; CON, control; MFI, mean fluorescence intensity; a.u., arbitrary units.

Given that TTFields have been associated with structural and metabolic stress responses in other cancer models, the present study assessed changes in intracellular complexity and mitochondrial-related parameters in T-ALL cells. SSC analysis revealed a consistent right-upward shift in FSC/SSC profiles following TTFields exposure, accompanied by a significant increase in median SSC-A values, indicating enhanced intracellular granularity (Fig. 3A). Consistent with these structural changes, MitoTracker green fluorescence staining demonstrated increased mitochondrial fluorescence intensity in both Jurkat and MOLT-4 cells (Fig. 3B). To assess whether these mitochondrial-associated changes were accompanied by alterations in cellular energy status, intracellular ATP levels were measured. TTFields-treated Jurkat cells exhibited a reduction in relative ATP levels, whereas no significant change was observed in MOLT-4 cells (Fig. 3C).

### TTFields modulate T-cell activation and immune-related transcription

**Figure 4:**
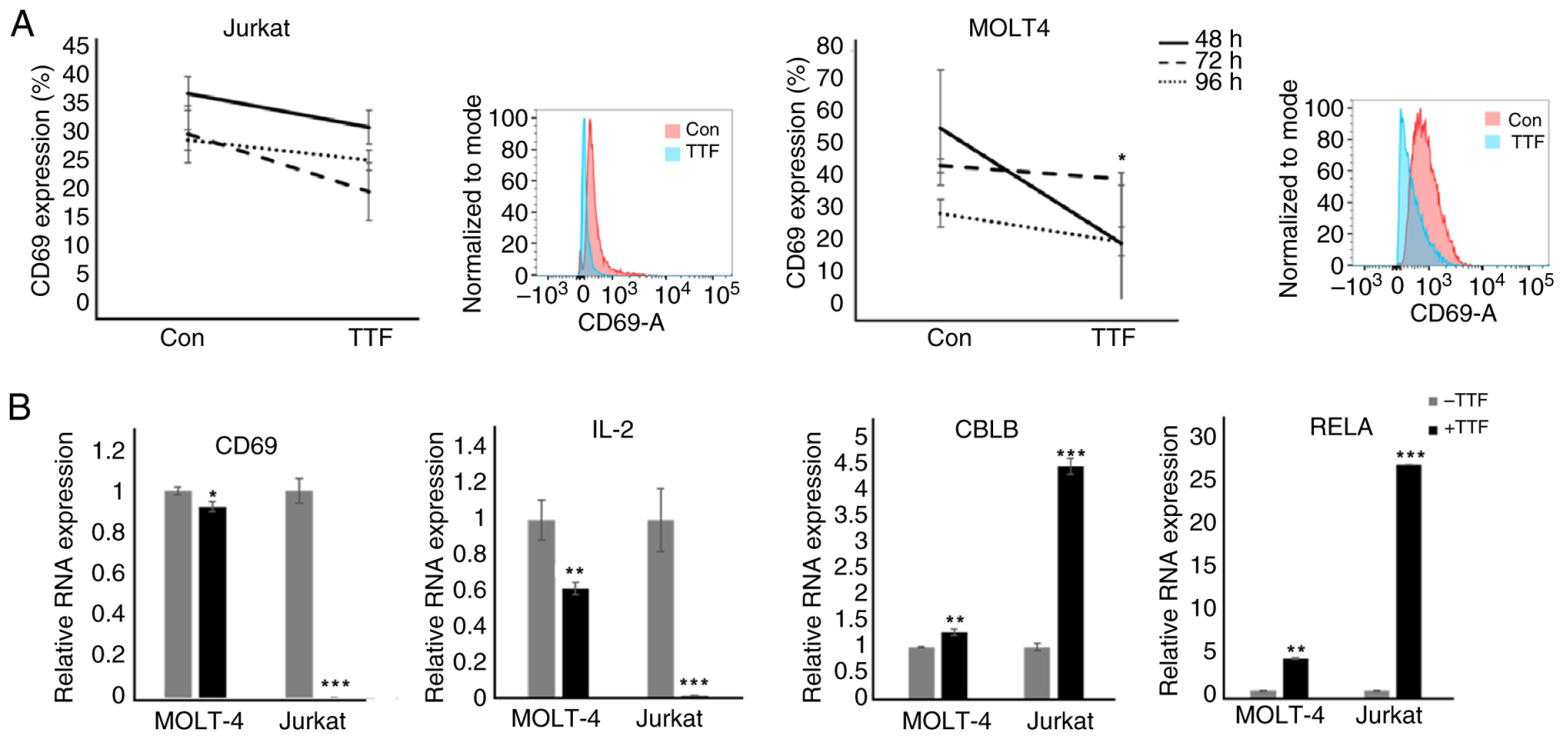
TTFields reduce CD69 expression and alter immune-related gene transcription in T-cell acute lymphoblastic leukemia cells. (A) Flow-cytometric analysis showed reduced CD69^+^ fractions in pre-activated Jurkat and MOLT-4 cells following 48-96 h TTFields exposure, with quantitative summaries indicating a consistent downward trend. TTFields treatment reduced CD69 expression in MOLT-4 cells, whereas no significant change was observed in Jurkat cells. (B) Gene-expression profiling after 48 h further demonstrated decreased CD69 and IL-2 and increased RELA and CBLB transcripts. Data represent the mean ± SD from three independent experiments. ^*^P<0.05, ^**^P<0.01 and ^***^P<0.001. TTFields/TTF, Tumor Treating Fields; RELA, RELA proto-oncogene, NF-κB subunit; CBLB, CBL proto-oncogene B; Con, control.

To assess whether TTFields influence T-cell activation programs, CD69 surface expression and immune-related gene transcription were evaluated in pre-activated Jurkat and MOLT-4 cells. Across 48-96 h TTFields exposure, both cell lines consistently showed reduced CD69 expression compared with activated controls, indicating attenuated activation signaling (Fig. 4A). qPCR profiling after 48 h further demonstrated downregulation of CD69 and IL-2 transcripts, along with significant upregulation of RELA and CBLB (particularly pronounced in Jurkat cells) suggesting NF-κB-associated adaptive reprogramming (Fig. 4B). These results collectively indicate that TTFields reshapes activation-associated signaling in leukemic T-cells at both phenotypic and transcriptional levels.

## Discussion

T-ALL is a highly aggressive hematological malignancy with poor prognosis and frequent relapse. Conventional chemotherapy often leads to resistance and severe systemic toxicity (12), underscoring the urgent need for alternative, non-drug-based therapeutic strategies. Owing to its capacity to physically disrupt cellular division and intracellular signaling, TTFields have emerged as a promising biophysical modality. While TTFields have demonstrated efficacy in a number of solid tumors (13-15), their application to hematologic malignancies remains largely unexplored. Accordingly, the present study aimed to elucidate the biological responses of T-ALL cells to TTFields and to assess their potential as an adjunctive therapeutic strategy in leukemia.

TTFields exposure in T-ALL cells led to progressive inhibition of cell proliferation and viability, indicating a distinct cytostatic mode of action compared with conventional chemotherapy. Flow cytometric and cell-cycle analyses further revealed increased late-apoptotic (Annexin V^+^/PI^+^) fractions, without a clearly defined phase-specific arrest and elevated sub-G_1_ populations, demonstrating that TTFields primarily induce apoptosis rather than a distinct cell-cycle arrest. These effects are consistent with previous reports describing TTFields-mediated microtubule disassembly, chromosomal segregation errors and cytokinesis failure (16-18). Notably, Jurkat cells exhibited stronger apoptotic and mitotic responses compared with MOLT-4 cells, suggesting that cell line-specific signaling and metabolic contexts may contribute to variable susceptibility to electric-field stimulation.

TTFields also induced coordinated immune-related transcriptional programs in leukemic T-cells. Cells exposed to TTFields exhibited a consistent reduction of the early activation marker CD69, accompanied by downregulation of CD69 and IL-2 transcripts. By contrast, RELA (NF-κB p65) and CBLB were markedly upregulated in Jurkat cells (by ~25-fold and 4.5-fold, respectively) and modestly increased in MOLT-4 cells. RELA activation may reflect a stress-associated survival response, whereas sustained NF-κB signaling could also contribute to apoptotic commitment. Upregulation of CBLB likely represents a feedback mechanism to restrain excessive TCR signaling (19-21). Notably, these findings were derived from transcriptional and activation-marker analyses and therefore reflect altered T-cell activation and regulatory signaling states rather than direct measurements of immune effector function. Functional validation using cytokine secretion assays, cytotoxicity measurements or co-culture systems is required to determine whether these signaling changes translate into altered immune effector activity.

In addition to transcriptional alterations, TTFields-treated cells exhibited increased side scatter, as reflected by elevated median side scatter-A values, indicating enhanced intracellular complexity. MitoTracker green fluorescence intensity was also increased, suggesting alterations in mitochondrial distribution or mass. However, these structural mitochondrial-associated changes were not uniformly associated with enhanced bioenergetic output, as intracellular ATP levels were reduced in Jurkat cells but remained unchanged in MOLT-4 cells. This dissociation suggests that TTFields-induced mitochondrial alterations may reflect stress-associated remodeling rather than uniform metabolic activation. Such structural alterations are consistent with organelle redistribution and metabolic stress reported in other TTFields-exposed models, including glioblastoma, lung cancer and mesothelioma cell-based systems, where TTFields have been shown to induce mitochondrial dysfunction, ATP depletion and autophagy-associated cellular remodeling (4,22,23). Notably, mitochondrial remodeling has been associated with early metabolic perturbations and redox imbalance (24,25), raising the possibility that TTFields-induced structural changes contribute to oxidative stress responses.

The differential sensitivity to TTFields between Jurkat and MOLT-4 cells may reflect intrinsic molecular and metabolic heterogeneity. MOLT-4 cells harbor activating NOTCH1 proline-glutamate-serine-threonine domain mutations, whereas Jurkat cells retain a wild-type NOTCH1 genotype, resulting in distinct basal NOTCH signaling contexts. Consistent with this, Jurkat and MOLT-4 cells have been reported to exhibit markedly different levels of active NOTCH1 intracellular domain, thereby differentially regulating downstream transcriptional and survival programs (26). In addition, metabolomic profiling has revealed clear baseline metabolic separation between these cell lines, even within the same TAL/LIM-only domain subgroup (27). Such signaling and metabolic differences may contribute to variability in TTFields responsiveness, warranting systematic baseline molecular profiling in future studies.

Despite these promising insights, the present study was limited to *in vitro* leukemic cell-line models and did not include comparisons with normal T-cells. Although TTFields showed minimal effects in non-malignant cells under the tested conditions, the lack of direct comparisons with primary normal T-cells or hematopoietic progenitors remains a limitation of the present study. Future investigations should therefore quantitatively assess reactive oxygen species production, mitochondrial membrane potential dynamics and downstream apoptotic cascades to further define TTFields-induced stress pathways. Integrating computational field simulations with biological experiments will be key in associating electric-field distribution with cellular responses. Moreover, co-culture or *in vivo* models incorporating normal hematopoietic and immune cells will be valuable in determining whether TTFields exert immunosuppressive or anti-leukemic effects. Finally, exploring combination regimens of TTFields with chemotherapy or immunotherapy could facilitate its translational development toward clinical application in leukemia.

## Supplementary Material

Minimal effects of TTFields on a non-malignant human fibroblast cell line. CCD-112CoN cells were exposed to TTFields under the same experimental conditions used for T-cell acute lymphoblastic leukemia cell lines. (A) Metabolic activity assessed by Water-Soluble Tetrazolium-8 (OD measured at 450 nm). (B) Cell number normalized to the untreated control (% of control). Data are presented as the mean ± SD from at least three independent experiments. Statistical significance was evaluated using an unpaired two-tailed Welch's t-test. ns, not significant; OD, optical density; TTFields/TTF, Tumor Treating Fields.

## Data Availability

The data generated in the present study may be requested from the corresponding author.
