# Supplementary material for: Tumor Treating Fields modulate apoptotic and immune programs in T-cell acute lymphoblastic leukemia cell lines
Source: Exp Ther Med. 2026 May 29;32(2):200. doi: 10.3892/etm.2026.13195 (PMC13267092; doi:10.3892/etm.2026.13195)

Figure S1. Minimal effects of TTFields on a non-malignant human fibroblast cell line. CCD-112CoN cells were exposed to TTFields under the same experimental conditions used for T-cell acute lymphoblastic leukemia cell lines. (A) Metabolic activity assessed by Water-Soluble Tetrazolium-8 (OD measured at 450 nm). (B) Cell number normalized to the untreated control (% of control). Data are presented as the mean ± SD from at least three independent experiments. Statistical significance was evaluated using an unpaired two-tailed Welch's t-test. ns, not significant; OD, optical density; TTFields/TTF, Tumor Treating Fields.

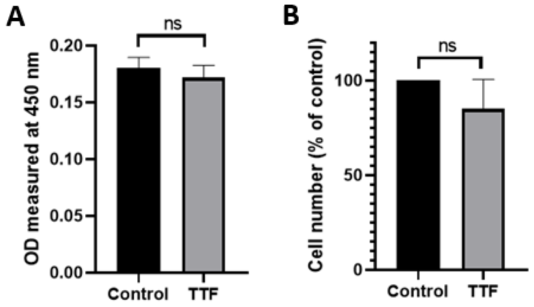

Supplement: Minimal effects of TTFields on a non-malignant human fibroblast cell line. CCD-112CoN cells were exposed to TTFields under the same experimental conditions used for T-cell acute lymphoblastic leukemia cell lines. (A) Metabolic activity assessed by Water-Soluble Tetrazolium-8 (OD measured at 450 nm). [file Supplementary_Data.pdf]
